# Adipocyte P2Y_14_ receptors play a key role in regulating whole-body glucose and lipid homeostasis

**DOI:** 10.1172/jci.insight.146577

**Published:** 2021-05-24

**Authors:** Shanu Jain, Sai P. Pydi, Young-Hwan Jung, Mirko Scortichini, Efrat L. Kesner, Tadeusz P. Karcz, Donald N. Cook, Oksana Gavrilova, Jürgen Wess, Kenneth A. Jacobson

**Affiliations:** 1 Molecular Recognition Section and; 2 Molecular Signaling Section, Laboratory of Bioorganic Chemistry, National Institute of Diabetes and Digestive and Kidney Diseases, Bethesda, Maryland, USA.; 3 Immunity, Inflammation, and Disease Laboratory, National Institute of Environmental Health Sciences, Research Triangle Park, North Carolina, USA.; 4 Mouse Metabolism Core, National Institute of Diabetes and Digestive and Kidney Diseases, Bethesda, Maryland, USA.

**Keywords:** Metabolism, Adipose tissue, Diabetes, Obesity

## Abstract

Obesity is the major driver of the worldwide epidemic in type 2 diabetes (T2D). In the obese state, chronically elevated plasma free fatty acid levels contribute to peripheral insulin resistance, which can ultimately lead to the development of T2D. For this reason, drugs that are able to regulate lipolytic processes in adipocytes are predicted to have considerable therapeutic potential. G_i_-coupled P2Y_14_ receptor (P2Y_14_R; endogenous agonist, UDP-glucose) is abundantly expressed in both mouse and human adipocytes. Because activated G_i_-type G proteins exert an antilipolytic effect, we explored the potential physiological relevance of adipocyte P2Y_14_Rs in regulating lipid and glucose homeostasis. Metabolic studies indicate that the lack of adipocyte P2Y_14_R enhanced lipolysis only in the fasting state, decreased body weight, and improved glucose tolerance and insulin sensitivity. Mechanistic studies suggested that adipocyte P2Y_14_R inhibits lipolysis by reducing lipolytic enzyme activity, including ATGL and HSL. In agreement with these findings, agonist treatment of control mice with a P2Y_14_R agonist decreased lipolysis, an effect that was sensitive to inhibition by a P2Y_14_R antagonist. In conclusion, we demonstrate that adipose P2Y_14_Rs were critical regulators of whole-body glucose and lipid homeostasis, suggesting that P2Y_14_R antagonists might be beneficial for the therapy of obesity and T2D.

## Introduction

Accumulation of excess nutrient energy as triacylglycerol (TAG) in adipocytes results in the development of obesity. Obesity is a key risk factor for the pathogenesis of insulin resistance and type 2 diabetes (T2D) and can aggravate metabolic complications such as nonalcoholic fatty liver disease and cardiovascular diseases (1). Therefore, identifying drug targets for improving the metabolic function of adipocytes is of utmost importance to prevent the metabolic deficits caused by obesity.

Net lipid storage in adipocytes and adipose tissue mass depends on a fine balance between anabolic and catabolic lipid pathways. Following nutrient intake, de novo fatty acid (FA) synthesis from glucose and amino acids is induced, and synthesis of TAG promotes lipid storage in adipocytes. Increased post-feeding circulating insulin levels inhibit TAG breakdown and lipolysis in adipocytes (2). In contrast, adipocyte lipolysis provides alternative energy substrates (FAs and glycerol) to other tissues during periods of caloric deprivation (e.g., fasting). Lipolysis is a highly regulated process that requires the activation of different lipases (2). Adipose triglyceride lipase (ATGL) and hormone-sensitive lipase (HSL) are the 2 major lipases converting TAG to diacylglycerol (DAG) and DAG to monoacylglycerol (MAG), respectively, with the liberation of a FA at each step (2). MAG is hydrolyzed to release the final FA and glycerol. The rate of basal lipolysis is increased in obesity resulting in elevated plasma FA levels that are predicted to contribute to the development of insulin resistance (3, 4). However, the activation of lipolysis by factors such as catecholamines activating β-adrenergic receptors is greatly impaired in obesity (5, 6).

Adipocyte metabolism is regulated by various GPCRs that are coupled to different functional classes of heterotrimeric G proteins (G_s_, G_q_, or G_i_) (7, 8). Purinergic (P2Y) receptors are a class of GPCRs that are activated by nucleotides and nucleotide sugars (9). In the obese state, high plasma levels of uracil nucleoside/nucleotides and nucleotide sugars have been reported to act on P2Y receptors (10, 11). G_i_-coupled receptors such as the CB_1_ cannabinoid receptor, GPR109A, and GPR81 are expressed in adipocytes and have been implicated in the regulation of adipocyte function such as lipolysis, insulin resistance, inflammation, and adipokine secretion (12–14). P2Y_14_R is a G_i_-coupled receptor activated by UDP-glucose (15, 16). P2Y_14_R is expressed in human tissues, including adipose tissue, stomach, intestine, placenta, and certain brain regions (17). P2Y_14_R is also markedly expressed in immune cells and plays a significant role in regulating innate immunity and inflammation (18–20). However, the contribution of adipocyte P2Y_14_R in the development of obesity and associated metabolic deficits remains unexplored.

To address this issue, we generated a mutant mouse model lacking P2Y_14_R specifically in adipocytes to examine the receptor’s role in adipocyte tissue physiology and its effect on whole-body glucose and energy homeostasis. Our data show that the lack of P2Y_14_R in adipocytes results in enhanced lipolysis during fasting conditions. This enhancement of lipolysis protected adipocyte-specific P2Y_14_R KO mice against diet-induced obesity (DIO), improving whole-body glucose and energy homeostasis. These findings suggest that P2Y_14_R may prove a useful target for the treatment of obesity and obesity-related metabolic disorders.

## Results

### P2Y_14_R expression level correlated with human obesity.

**Figure 1:**
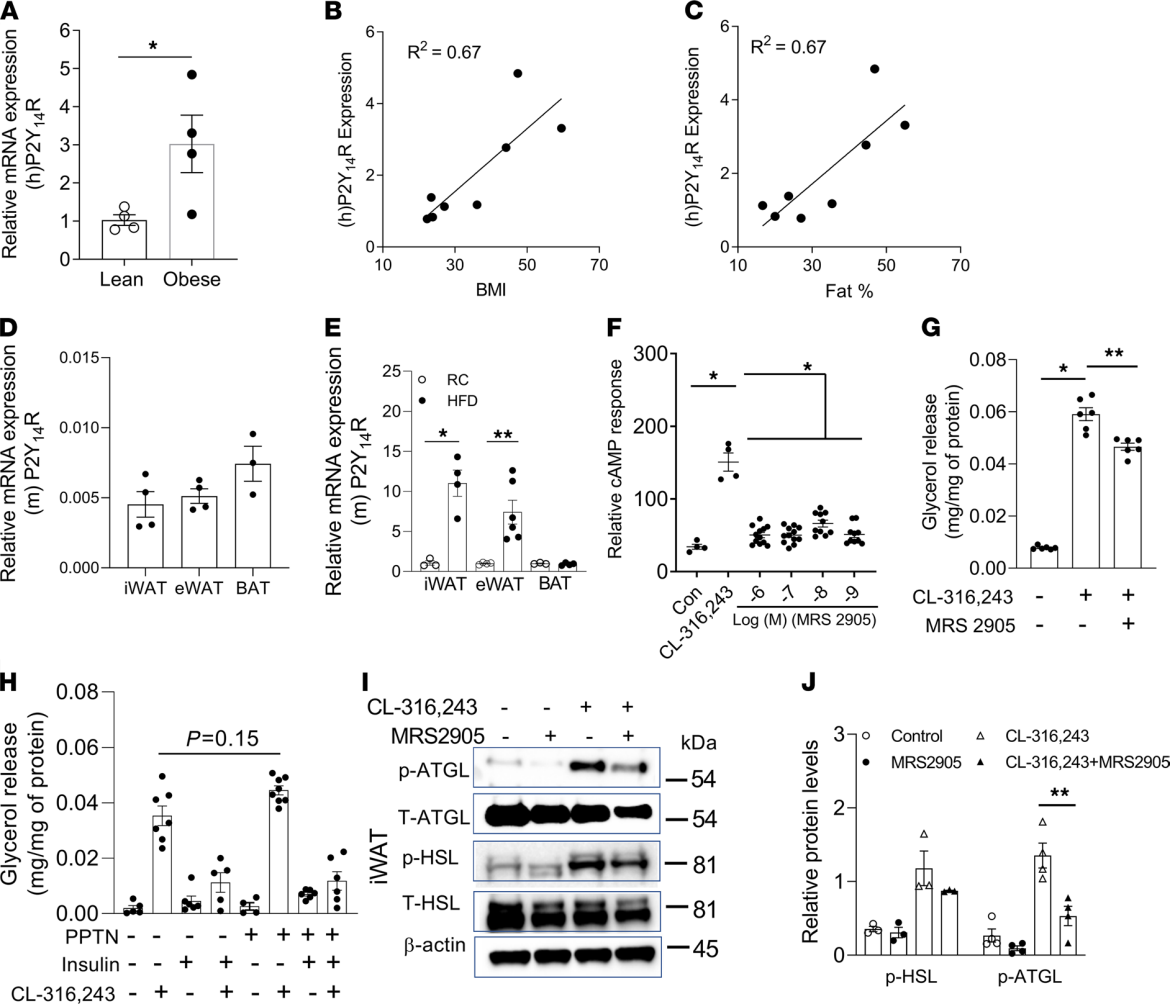
P2Y_14_R mRNA in human subcutaneous fat; activation of P2Y_14_R inhibits lipolysis in vitro. (**A**) Relative P2Y_14_R mRNA expression in subcutaneous fat from lean and obese humans (*n* = 3–4/group). (**B**) P2Y_14_ R mRNA and BMI correlation in lean and obese humans (*n* = 8). (**C**) P2Y_14_ R mRNA and fat mass (%) correlation in lean and obese human individuals (*n* = 8). (**D**) Relative expression of P2Y_14_ R mRNA in subcutaneous (iWAT), visceral (eWAT), and brown (BAT) mouse adipose tissues (*n* = 3–4/group). (**E**) P2Y_14_ R mRNA levels in iWAT, eWAT (mature adipocytes), and BAT from C57BL/6 control mice (*n* = 3 or 4/group) on RC or HFD. (**F**) Relative cAMP response in differentiated mature adipocytes (iWAT) from control mice, treated with CL-316243 (10 nM) (β_3_-adrenergic receptor-selective agonist) or MRS2905 with CL-316243 (10 nM), as indicated (*n* = 4/group). (**G**) Glycerol release in differentiated mature adipocytes (iWAT) from control mice stimulated with MRS2905 (2 nM) for 30 min. Cells were then treated with CL-316243 (10 nM), as indicated. (*n* = 3/group). (**H**) Glycerol release in differentiated mature adipocytes (iWAT) treated with vehicle or PPTN (200 nM) for 30 min, followed by insulin (10 nM) and/or CL-316,243 (10 nM) (*n* = 3/group). (**I**) Western blot analysis of p-ATGL/T-ATGL and p-HSL/T-HSL protein expression in differentiated mature adipocytes (iWAT, control mice) and treated with P2Y_14_R agonist (MRS2905, 2 nM) for 30 min, followed by CL-316243 (10 nM) for 30 min, as indicated. Representative blots are shown (*n* = 3 or 4 independent experiments). (**J**) Quantification of immunoblotting data shown in **I**. The expression of 18s rRNA was used to normalize qRT-PCR data. All data are expressed as mean ± SEM. *P < 0.05, **P < 0.01 (**A**–**E** and **J**: 2-tailed Student’s *t* test; **F**–**H**: 1-way ANOVA followed by Bonferroni’s post hoc test). All preadipocytes were isolated from RC-fed mice and differentiated to mature adipocytes for experiments. P2Y, purinergic; RC, regular chow; HFD, high-fat diet; ATGL, adipose triglyceride lipase; HSL, hormone-sensitive lipase.

P2Y_14_R is expressed in human adipose tissue (17). We first examined whether the P2Y_14_R expression level was altered in subcutaneous fat of obese human subjects suffering from insulin resistance compared with individuals (age-, race-, and sex-matched) without obesity and insulin resistance. Interestingly, P2Y_14_R expression levels were significantly increased in fat tissues of obese compared with the lean subjects (Figure 1A). Linear regression analysis showed a correlation between P2Y_14_R expression levels and BMI of human subjects under study (Figure 1B). P2Y_14_R expression was also correlated with fat (%) of lean and obese human subjects (Figure 1C). These data suggest that human P2Y_14_R may have played a critical role in the adipocyte biology and development of obesity and associated pathophysiological deficits.

### P2Y_14_R expression was regulated by high-fat diet in mouse adipocytes.

To determine the role of P2Y_14_R in adipose tissue physiology, we first quantified P2Y_14_R mRNA expression levels in different mouse adipose depots. RT-PCR analysis revealed that P2Y_14_R had similar expression levels in subcutaneous (iWAT), visceral (eWAT), and brown (BAT) adipose tissues (Figure 1D). We next determined whether consumption of a high-fat diet (HFD) regulates P2Y_14_R expression levels in mouse adipocytes. Interestingly, P2Y_14_R mRNA levels were upregulated in isolated mature adipocytes from iWAT and eWAT but not BAT of HFD mice (Figure 1E). The upregulation of P2Y_14_R expression in WAT of HFD mice may indicate that P2Y_14_R signaling played a role in regulating WAT function during obesity.

### P2Y_14_R activation inhibited lipolysis in mature adipocytes.

Many GPCRs mediate lipolytic or antilipolytic effects through the regulation of adipocyte cAMP levels (21). Because P2Y_14_R couples to G_i_-type G proteins (15), we next studied the effect of P2Y_14_R activation on cAMP levels in mouse adipocytes. Preadipocytes from mouse iWAT were isolated and differentiated into mature adipocytes to study adipocyte lipolysis. As expected, P2Y_14_R activation by MRS2905, a selective P2Y_14_R agonist (22), inhibited the increase in cAMP accumulation caused by treatment with CL-316,243, a β_3_-adrenergic receptor agonist (% reduction by 1 nM MRS2905: 66.04 ± 3.93) (Figure 1F). Similarly, stimulation with MRS2905 decreased CL-316,243-induced glycerol release in differentiated mouse mature adipocytes (% reduction: 21.02 ± 3.24) (Figure 1G). Further, cells were treated with PPTN (P2Y_14_R antagonist, 200 nM, 30 min) (23), and the effect of adrenergic stimulation with or without insulin on lipolysis was studied. Blockade of P2Y_14_R with PPTN showed a trend toward enhanced CL-316,243-mediated lipolysis (% increase: 25.96 ± 6.47), possibly due to increased cAMP level in cells (Figure 1H). Acute stimulation with insulin decreased CL-316,243-mediated lipolysis to similar levels in both control and PPTN-treated cells, indicating insulin-independent regulation of lipolysis by P2Y_14_R (Figure 1H). Mechanistically, CL-316,243-induced phosphorylation of ATGL was decreased by P2Y_14_R activation in differentiated mature adipocytes (iWAT; % reduction: 60.67 ± 13.77; Figure 1, I and J). Together, these results suggest that the activation of P2Y_14_R inhibited lipolysis in differentiated mature adipocytes in vitro.

### Acute activation of P2Y_14_R decreased lipolysis in vivo.

**Figure 2:**
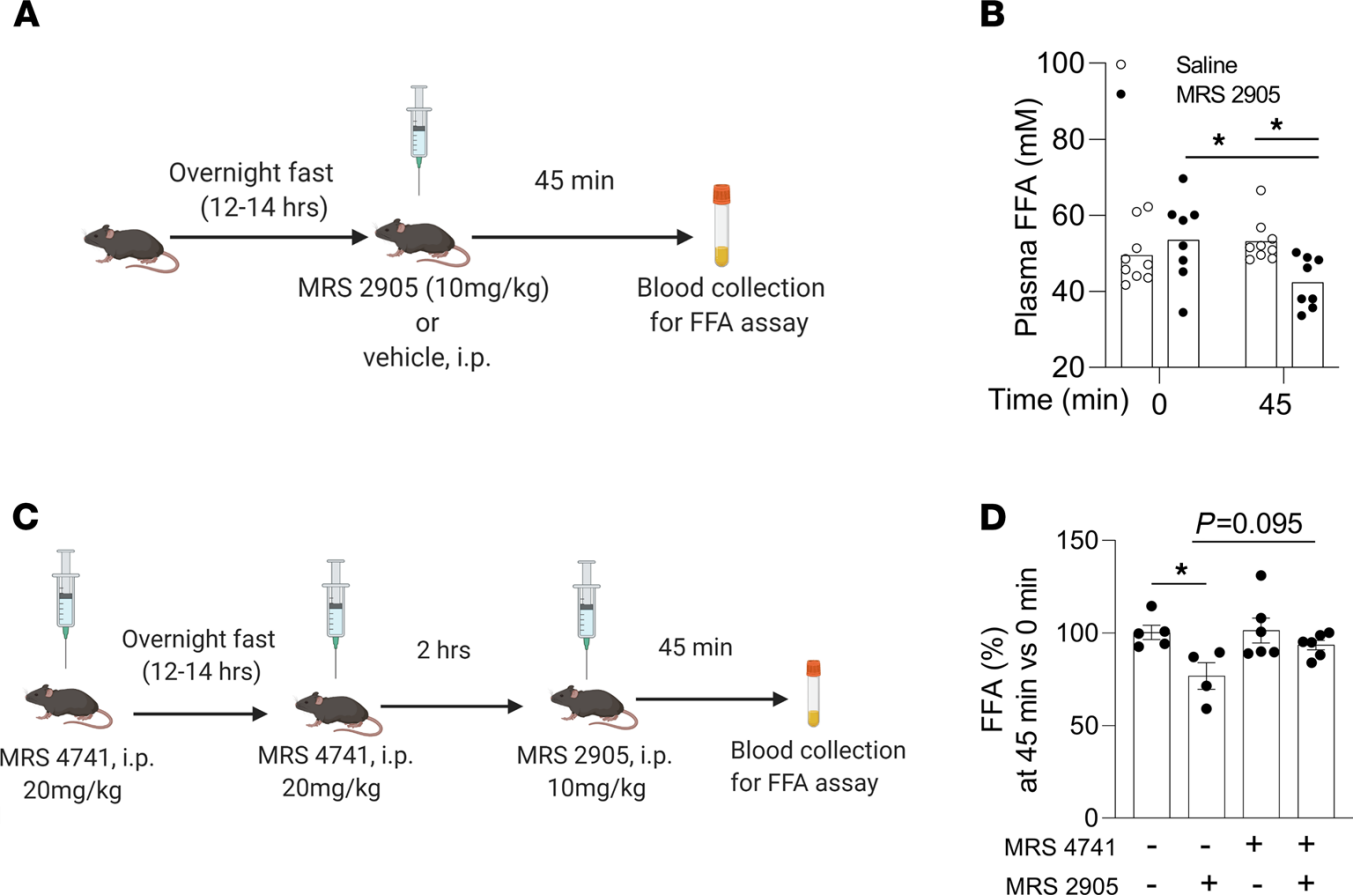
Activation of P2Y_14_R inhibits lipolysis in mice. (**A**) Schematic representation of timeline for MRS2905 or vehicle injection (i.p.) in overnight fasted mice and blood collection for FFA assay. (**B**) Plasma FFA levels in HFD WT mice i.p. injected with saline or MRS2905 (10 mg/kg, i.p.). Blood was collected immediately before and 45 min after injections (*n* = 8 or 9/group). (**C**) Schematic representation of timeline for treatment of mice with vehicle or MRS4741 (20 mg/kg, i.p.). Mice were then injected with vehicle or MRS2905 (10 mg/kg, i.p.) as indicated. Blood was collected at indicated time points for FFA assay. (**D**) Plasma FFA (%) in control mice treated with vehicle or MRS4741 (20 mg/kg, i.p.). Mice were then injected with vehicle or MRS2905 (10 mg/kg, i.p.) as indicated. Blood was collected immediately before and 45 min after MRS2905 injections (*n* = 4–6/group). All data are expressed as mean ± SEM. *P < 0.05 (1-way ANOVA followed by Bonferroni’s post hoc test). All agonist/antagonist injections were conducted on WT mice maintained on an HFD for at least 8 weeks. Graphics created using Biorender.com. P2Y, purinergic; FFA, free fatty acid; HFD, high-fat diet.

To examine whether the acute activation of P2Y_14_R was able to regulate lipolysis, we injected fasted (overnight) HFD control mice with P2Y_14_R agonist (MRS2905, 10 mg/kg, i.p.) or saline (Figure 2A). MRS2905 treatment resulted in a significant reduction in plasma free fatty acid (FFA) levels 45 minutes after injections, compared with the saline-treated group (FFA levels [mM] at 45 minutes after i.p.: saline [53.15 ± 2.67]; MRS2905 in saline [42.40 ± 3.33]; Figure 2B). Blocking P2Y_14_R with the antagonist prodrug MRS4741 (24) showed a trend toward rescuing the decrease in plasma FFA levels caused by the activation of P2Y_14_R by MRS2905 (Figure 2, C and D). In summary, acute activation of P2Y_14_R decreased lipolysis in vivo, resulting in reduced plasma FFA levels.

### Lack of adipocyte P2Y_14_R protected against HFD-induced obesity and improves metabolism.

**Figure 3:**
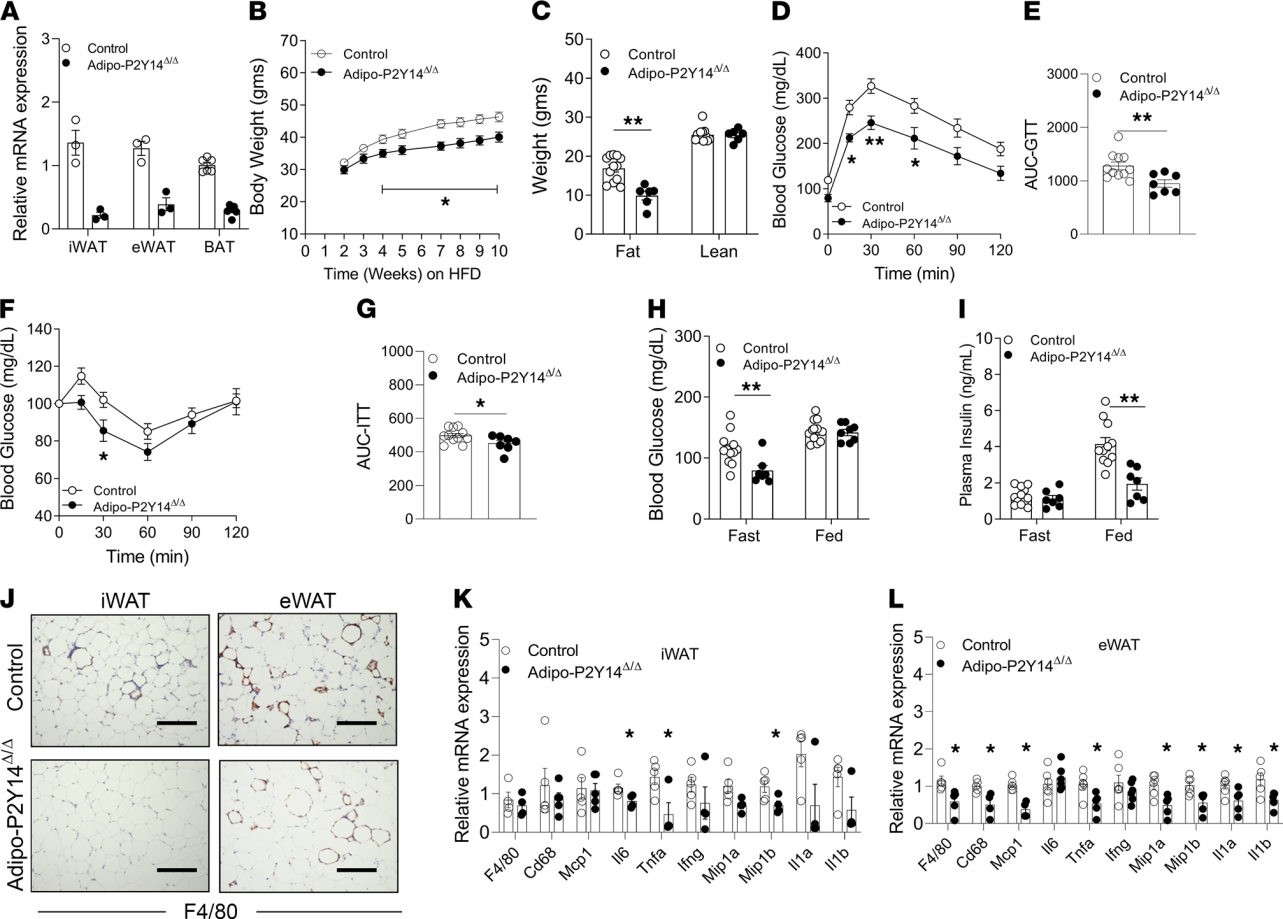
Adipocyte-specific P2Y_14_R KO mice (adipo-P2Y_14_^Δ/Δ^) are protected from DIO, inflammation and obesity-linked metabolic deficits. (**A**) mRNA expression levels of P2Y_14_R in mature adipocytes isolated from iWAT (*n* = 3/group), eWAT (*n* = 3/group), and BAT (*n* = 5/group) of HFD adipo-P2Y_14_^Δ/Δ^ and control mice. (**B**) Body weight measurements of mice maintained on HFD (*n* = 8 or 9/group). (**C**) Body composition (lean and fat mass) of mice maintained on HFD (*n* = 6–10/group). (**D**) GTT (1 g /kg glucose, i.p.) (*n* = 7–11/group). (**E**) AUC for **D**. (**F**) ITT (1 U/kg insulin, i.p.) (*n* = 7–11/group). (**G**) AUC for **F**. (**H**) Fasting and fed blood glucose levels (*n* = 7–11/group). (**I**) Fasting and fed plasma insulin levels (*n* *=* 7–11/group). (**J**) Representative F4/80-stained sections of iWAT and eWAT from HFD adipo-P2Y_14_^Δ/Δ^ and control mice. (**K**) Relative mRNA expression levels of inflammatory genes in iWAT from HFD adipo-P2Y_14_^Δ/Δ^ and control mice (*n* = 4 or 5/group). (**L**) Relative mRNA expression levels of inflammatory genes in eWAT from HFD adipo-P2Y_14_^Δ/Δ^ and control mice (*n* = 4–6/group). The expression of 18s rRNA was used to normalize qRT-PCR data. All data are expressed as mean ± SEM. *P < 0.05, **P < 0.01 (**A**, **C**, **E**, and **G**–**L**: 2-tailed Student’s *t* test; **B**, **D**, and **F**: 2-way ANOVA followed by Bonferroni’s post hoc test). All experiments were conducted on mice maintained on an HFD for at least 8 weeks. Scale bar: 150 µm. P2Y, purinergic; DIO, diet-induced obesity; HFD, high-fat diet; GTT, glucose tolerance test; ITT, insulin tolerance test.

Impaired regulation of lipolysis is associated with the development of obesity and insulin resistance (2). Because P2Y_14_R activation inhibits lipolysis, we next examined the physiological relevance of this phenomenon on whole-body glucose and energy homeostasis. To this end, we generated a mouse model lacking P2Y_14_R specifically in adipocytes (adipo-P2Y_14_^Δ/Δ^ mice) (Figure 3A and Supplemental Figure 1A; supplemental material available online with this article; https://doi.org/10.1172/jci.insight.146577DS1). Adipo-P2Y_14_^Δ/Δ^ mice and their control littermates (P2Y_14_*^fl/fl^*) were maintained on regular mouse chow (RC) or an HFD. On RC, adipo-P2Y_14_^Δ/Δ^ and control mice did not exhibit any significant differences in body weight or fat and lean mass, as determined by an MRI scan (Supplemental Figure 1, B and C). Similarly, adipo-P2Y_14_^Δ/Δ^ and control mice consuming RC displayed no differences in glucose tolerance, insulin sensitivity, fed and fasted blood glucose levels, or plasma insulin levels (Supplemental Figure 1, D and E, and Supplemental Figure 2, A and B). Fasted adipo-P2Y_14_^Δ/Δ^ mice showed a trend toward higher plasma FFA levels compared with fasted RC control mice, although this difference did not reach statistical significance (*P* = 0.18; Supplemental Figure 2C). No differences in plasma FFA levels were found in the fed state between the 2 mouse groups (Supplemental Figure 2C). Circulating levels of adipokines such as leptin and adiponectin were similar between the groups on RC (Supplemental Figure 2, D and E).

In striking contrast, adipo-P2Y_14_^Δ/Δ^ mice maintained on an HFD gained less weight than control mice (Figure 3B). MRI analysis revealed that the difference in body weight was due to a decrease in fat mass in adipo-P2Y_14_^Δ/Δ^ mice (Figure 3C). No difference in lean mass was observed between the 2 groups (Figure 3C). H&E staining revealed reduced adipocyte size (iWAT and eWAT) in adipo-P2Y_14_^Δ/Δ^ mice, compared with HFD control mice (Supplemental Figure 3). Moreover, HFD adipo-P2Y_14_^Δ/Δ^ mice showed significantly improved glucose tolerance and insulin sensitivity, most likely due to reduced adiposity (Figure 3, D–G). HFD adipo-P2Y_14_^Δ/Δ^ mice also displayed significantly decreased fasting blood glucose and fed plasma insulin levels, indicative of improved whole-body glucose homeostasis (Figure 3, H and I). Plasma leptin levels were decreased in the fasted state in HFD adipo-P2Y_14_^Δ/Δ^ mice (Supplemental Figure 4A), whereas adiponectin levels were increased in both fasted and fed states (Supplemental Figure 4B). Consistently, mRNA levels of leptin were decreased in both iWAT and eWAT of HFD adipo-P2Y_14_^Δ/Δ^ mice (Supplemental Figure 4C). mRNA levels of adiponectin were increased in eWAT of HFD adipo-P2Y_14_^Δ/Δ^ mice (Supplemental Figure 4D). Further, the acute activation of P2Y_14_R also decreased circulating levels of adiponectin (Supplemental Figure 4E), suggesting P2Y_14_R-mediated regulation of adiponectin secretion.

Moreover, the lack of P2Y_14_R decreased inflammation in adipose tissues of HFD adipo-P2Y_14_^Δ/Δ^ mice (Figure 3, J–L, and Supplemental Figure 5). F4/80 staining and mRNA levels of macrophage markers (f4/80 and cd68) were significantly reduced in eWAT of HFD adipo-P2Y_14_^Δ/Δ^ mice (Figure 3, J and L), and iWAT of HFD adipo-P2Y_14_^Δ/Δ^ mice displayed reduced mRNA levels of the inflammation markers IL-6, TNF-α, and MIP1β (Figure 3, J and K). Reduced mRNA of inflammatory markers was also observed in BAT of adipo-P2Y_14_^Δ/Δ^ mice (Supplemental Figure 5).

### Adipocyte-specific P2Y_14_R mutant mice exhibit increased fasting lipolysis.

**Figure 4:**
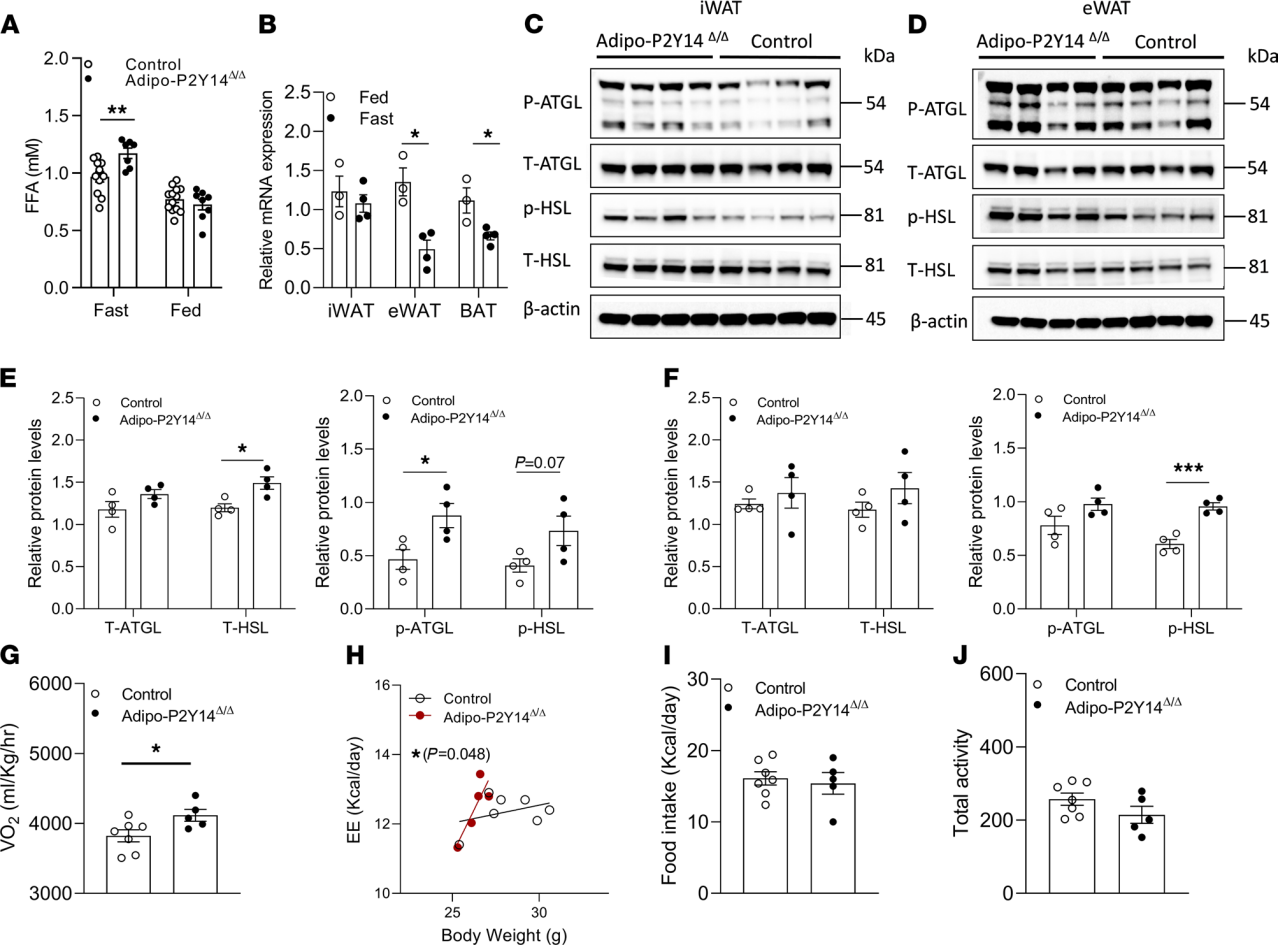
Adipo-P2Y_14_^Δ/Δ^ mice exhibit enhanced lipolysis under fasting conditions. (**A**) Fasting and fed plasma FFA levels of HFD mice (*n* = 7–11/group). (**B**) mRNA expression levels of P2Y_14_R in iWAT, eWAT, and BAT of 12-hour fasted and fed HFD control mice (*n* = 3 or 4/group). (**C**) Western blots showing increased T-ATGL, p-ATGL, T-HSL, and p-HSL expression in iWAT of fasted HFD adipo-P2Y_14_^Δ/Δ^ and control mice. Each lane represents a different mouse (*n* = 4/group). (**D**) Western blots showing increased T-ATGL, p-ATGL, T-HSL, and p-HSL expression in eWAT of fasted HFD adipo-P2Y_14_^Δ/Δ^ and control mice. Each lane represents a different mouse (*n* = 4/group). (**E**) Quantification of immunoblotting data shown in **C**. (**F**) Quantification of immunoblotting data shown in **D**. (**G**) Oxygen consumption rate (VO_2_) (*n* = 5–7/group). (**H**) Total energy expenditure normalized to body weight (*n* = 5–7/group). (**I**) Food intake (*n* = 5–7/group). (**J**) Total locomotor activity (*n* = 5–7/group). The expression of 18s rRNA was used to normalize qRT-PCR data. All data are expressed as mean ± SEM. *P < 0.05, **P < 0.01, ***P < 0.001 (**A**–**G**, **I** and **J**: 2-tailed Student’s *t* test; **H**: ANCOVA analysis). All experiments were conducted on mice maintained on HFD for at least 8 weeks. P2Y, purinergic; FFA, free fatty acid; HFD, high-fat diet; ATGL, adipose triglyceride lipase; HSL, hormone-sensitive lipase.

We next examined the contribution of P2Y_14_R to adipocyte lipolysis in vivo. Specifically, we first checked plasma FFA levels in HFD adipo-P2Y_14_^Δ/Δ^ and control mice. HFD Adipo-P2Y_14_^Δ/Δ^ mice displayed elevated plasma FFA levels after a 12-hour fast (FFA [mM] — control: [0.94 ± 0.07], adipo-P2Y_14_^Δ/Δ^ [1.16 ± 0.05]; Figure 4A). In contrast, no difference in plasma FFA levels was observed between the 2 groups under fed conditions (Figure 4A). Fasting also caused a significant decrease in P2Y_14_R mRNA expression in eWAT and BAT of HFD mice (Figure 4B). These data indicate that the lack of P2Y_14_R in adipocytes acutely increased lipolysis to provide energy substrates to other organs during nutritional deficient conditions. This enhancement in lipolysis during fasting may have contributed to the reduced fat mass observed with HFD adipo-P2Y_14_^Δ/Δ^ mice.

Next, to decipher the mechanism by which P2Y_14_R regulates lipolysis, we analyzed the activity of HSL and ATGL in adipose depots of fasted HFD adipo-P2Y_14_^Δ/Δ^ mice and control mice. The expression levels of total HSL (T-HSL) were significantly enhanced in iWAT of fasted adipo-P2Y_14_^Δ/Δ^ mice (Figure 4, C and E). The expression of the activated form of HSL (p-HSL; S563) tended to be increased (*P* = 0.07) in iWAT of fasted HFD adipo-P2Y_14_^Δ/Δ^ mice (Figure 4, C and E). On the other hand, the expression levels of total ATGL (T-ATGL) were not significantly different between mutant and control mice (iWAT; Figure 4, C and E). However, the expression of an activated form of ATGL (p-ATGL; S406) was significantly enhanced in iWAT of fasted HFD adipo-P2Y_14_^Δ/Δ^ mice (Figure 4, C and E). In eWAT, no difference was observed in T-HSL and T-ATGL between mutant and control mice (Figure 4, D and F). The expression levels of p-HSL were significantly enhanced in eWAT of fasted HFD adipo-P2Y_14_^Δ/Δ^ mice (Figure 4, D and F). On the other hand, no significant difference in ATGL phosphorylation was observed in eWAT of fasted mice (Figure 4, D and F). These data indicate that P2Y_14_R KO enhances lipolysis in nutrient-deficient conditions by increasing the activity of HSL and ATGL enzymes in WAT.

### Enhanced energy expenditure in HFD adipo-P2Y_14_**^Δ/Δ^ mice.

Lipolysis is essential for energy homeostasis in mice (25, 26). The observation that P2Y_14_R regulates cAMP levels and lipolysis in adipocytes led us to examine the role of adipose P2Y_14_R on energy homeostasis. Adipo-P2Y_14_^Δ/Δ^ and control mice were subjected to indirect calorimetry analysis during regular diet and the first week of HFD feeding when the 2 mouse groups did not differ significantly in body weight. The effects of body weight and body composition on energy expenditure (EE) was normalized by plotting EE to BW (or fat/lean body mass) (27). ANCOVA analysis revealed no difference in EE between regular diet fed control and adipo-P2Y_14_^Δ/Δ^ mice (Supplemental Figure 6, A–C). Oxygen consumption (VO_2_) and EE (normalized to BW) were modestly but significantly increased in HFD adipo-P2Y_14_^Δ/Δ^ mice (Figure 4, G and H). Normalizing EE to fat mass in HFD control and adipo-P2Y_14_^Δ/Δ^ mice did not show significant difference in EE between the groups (Supplemental Figure 6D), whereas normalizing EE to lean mass showed a trend toward enhanced EE in adipo-P2Y_14_^Δ/Δ^ mice (Supplemental Figure 6E). No differences in food intake and locomotor activity were observed between the 2 groups of mice (Figure 4, I and J). These data suggest that the increased availability of FFA due to enhanced lipolysis in adipo-P2Y_14_^Δ/Δ^ mice may have caused increased EE and most likely resulted in reduced adiposity displayed by HFD adipo-P2Y_14_^Δ/Δ^ mice.

### HFD adipo-P2Y_14_^Δ/Δ^ mice were protected from hepatic steatosis and insulin resistance.

**Figure 5:**
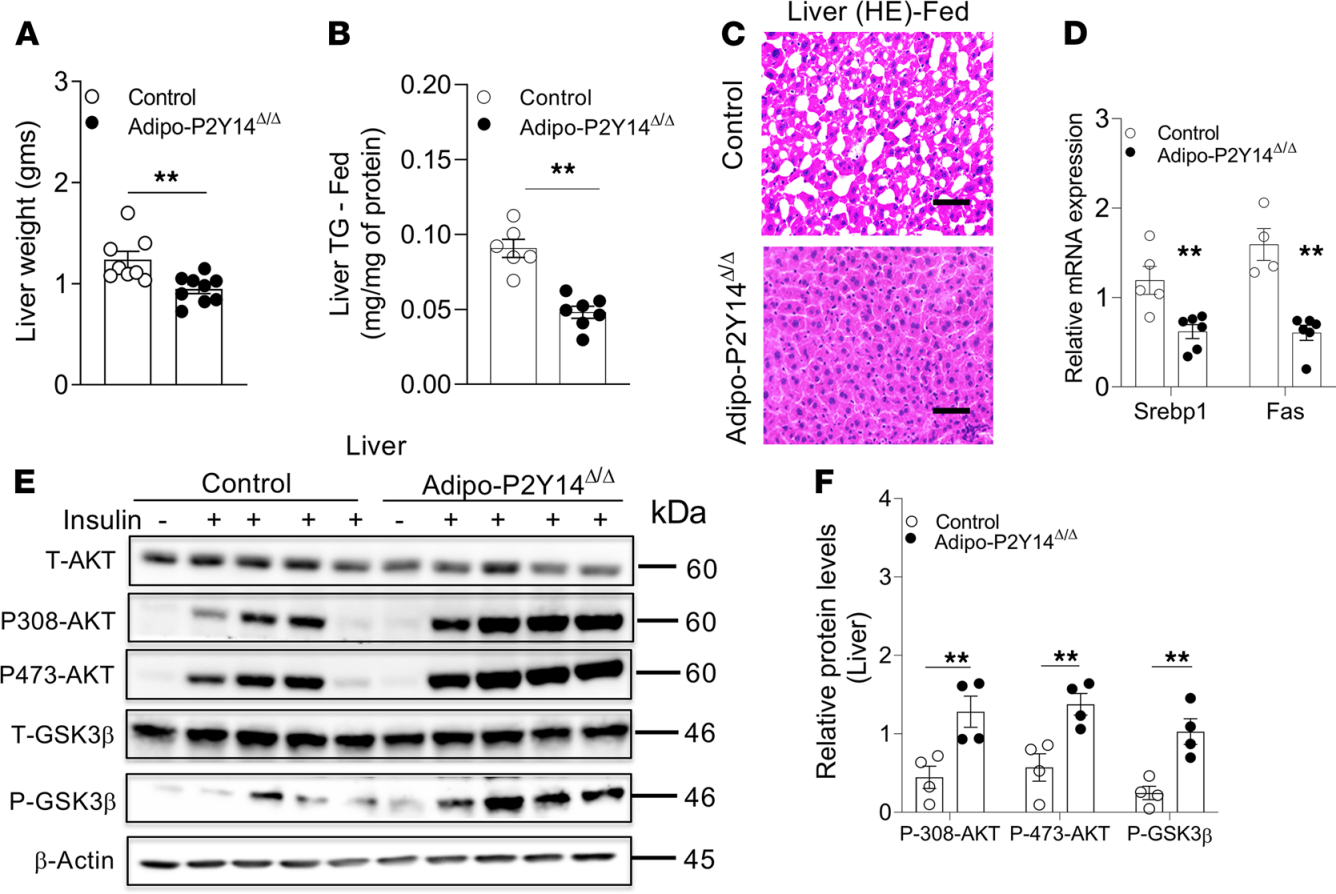
Adipo-P2Y_14_^Δ/Δ^ mice show protection from liver steatosis and improved hepatic insulin sensitivity. (**A**) Liver weight (grams) from adipo-P2Y_14_^Δ/Δ^ and control mice (*n* = 8 or 9/group). (**B**) Liver triglyceride (TG) levels from adipo-P2Y_14_^Δ/Δ^ and control mice (*n* = 6–7/group). (**C**) Representative H&E-stained sections of liver from HFD adipo-P2Y_14_^Δ/Δ^ and control mice. (**D**) Gene expression levels of *Srebp1* and *Fas* in liver of HFD adipo-P2Y_14_^Δ/Δ^ and control mice (*n* = 4–6/group). (**E**) Western blot analysis of insulin signaling in liver of HFD adipo-P2Y_14_^Δ/Δ^ and control mice (*n* = 4/group). (**F**) Quantification of immunoblotting data for **E** (*n* = 4/group). The expression of 18s rRNA was used to normalize qRT-PCR data. All data are expressed as mean ± SEM. **P < 0.01 (2-tailed Student’s *t* test). All experiments were conducted on mice consuming an HFD for at least 8 weeks. Scale bar: 150 µm. P2Y, purinergic; HFD, high-fat diet.

Increased plasma FFA levels are associated with the development of hepatic steatosis and insulin resistance. Because fasting FFA levels were increased in adipo-P2Y_14_^Δ/Δ^ mice, we next studied whether HFD adipo-P2Y_14_^Δ/Δ^ mice develop liver steatosis and insulin resistance. Surprisingly, HFD adipo-P2Y_14_^Δ/Δ^ mice displayed decreased liver weight, compared with HFD control mice (Figure 5A). In agreement with this observation, HFD adipo-P2Y_14_^Δ/Δ^ mice showed a significant decrease in liver triglyceride content in both fed and fasted conditions (Figure 5B and Supplemental Figure 7A). Decreased liver steatosis was further confirmed by H&E staining of liver sections. The liver sections from fed and fasted adipo-P2Y_14_^Δ/Δ^ mice displayed reduced lipid deposition than liver sections from control mice (Figure 5C and Supplemental Figure 7B). Oil red O staining of liver sections from fasted mice confirmed reduced lipid deposition in adipo-P2Y_14_^Δ/Δ^ compared with the control mice (Supplemental Figure 7B). The mRNA levels of genes involved in hepatic triglyceride accumulation, including *Srebp1* and *Fas,* were also reduced in HFD adipo-P2Y_14_^Δ/Δ^ mice (Figure 5D). To study insulin signaling in hepatic tissue, we injected HFD adipo-P2Y_14_^Δ/Δ^ and control mice with saline or insulin (5 U/mouse, i.v.) and collected liver tissues 5 minutes later. Immunoblotting studies indicated enhanced phosphorylation of AKT and GSK3β in the liver of HFD adipo-P2Y_14_^Δ/Δ^ mice (Figure 5, E and F). In summary, these data indicate that the lack of P2Y_14_R in adipocytes protected mice from the development of liver steatosis and hepatic insulin resistance. Reduced obesity and enhanced adiponectin levels may have contributed to improved liver function observed in HFD adipo-P2Y_14_^Δ/Δ^ mice.

### Lack of P2Y_14_R improved adipose tissue insulin sensitivity.

**Figure 6:**
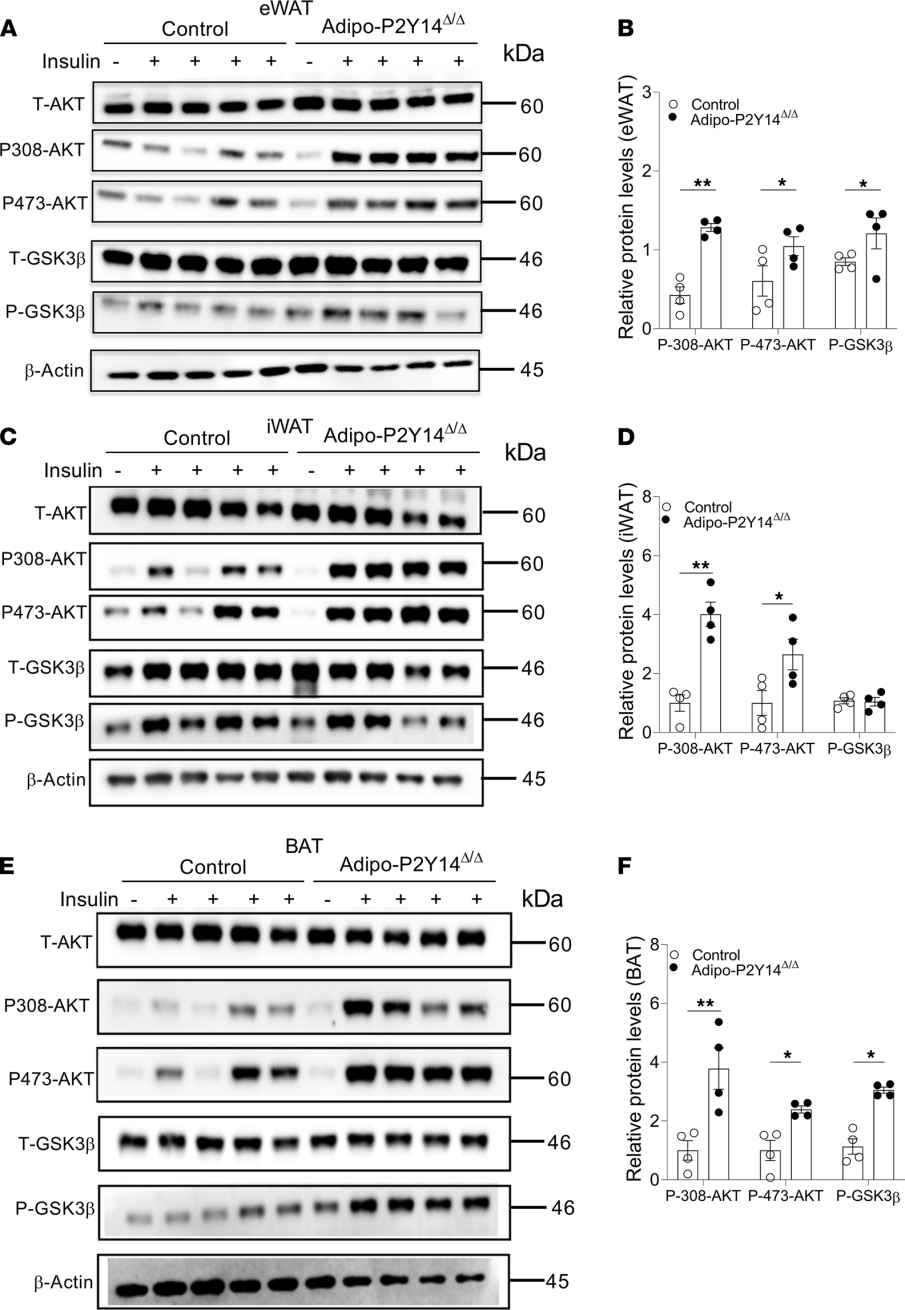
Adipo-P2Y_14_^Δ/Δ^ mice show improved adipose insulin sensitivity. (**A**) Western blot analysis of insulin signaling in eWAT of HFD adipo-P2Y_14_^Δ/Δ^ and control mice (*n* = 4/group). (**B**) Quantification of immunoblotting data for **A** (*n* = 4/group). (**C**) Western blot analysis of insulin signaling in iWAT of HFD adipo-P2Y_14_^Δ/Δ^ and control mice (*n* = 4/group). (**D**) Quantification of immunoblotting data for **C** (*n* = 4/group). (**E**) Western blot analysis of insulin signaling in BAT of HFD adipo-P2Y_14_^Δ/Δ^ and control mice (*n* = 4/group). (**F**) Quantification of immunoblotting data for (**E**) (*n* = 4/group). All data are expressed as mean ± SEM. *P < 0.05, **P < 0.01 (2-tailed Student’s *t* test). All experiments were conducted on mice consuming an HFD for at least 8 weeks. P2Y, purinergic; HFD, high-fat diet.

We next analyzed the effect of adipocyte P2Y_14_R deficiency on insulin sensitivity of different adipose tissues. To this end, we injected HFD adipo-P2Y_14_^Δ/Δ^ and control mice with saline or insulin (5 U/mouse, i.v.) and collected eWAT, iWAT and BAT 10 minutes later. Western blotting studies showed that the lack of P2Y_14_R in eWAT enhanced insulin-induced phosphorylation of AKT and GSK3β, suggesting improved insulin sensitivity (Figure 6, A and B). Enhanced phosphorylation of AKT was also observed in iWAT from HFD adipo-P2Y_14_^Δ/Δ^ mice (Figure 6, C and D). Similarly, phosphorylation of AKT and GSK3β was increased in BAT of HFD adipo-P2Y_14_^Δ/Δ^ mice (Figure 6, E and F). Together, these results indicate that the lack of P2Y_14_R in adipocytes improved insulin sensitivity in iWAT, eWAT, and BAT in mice consuming an HFD.

## Discussion

Obesity is associated with an increase in basal lipolysis and elevated plasma FFA levels (3, 4). Chronic unrestrained lipolysis mediates the development of insulin resistance, T2D, and fatty liver disease (28–30). Restrained lipolysis has been shown to protect against DIO by enhancing FA oxidation and energy expenditure (31–33). In this study, we identified a P2Y_14_R-mediated regulation of adipocyte lipolysis. We demonstrated that the inhibition of P2Y_14_R signaling in adipocytes increases lipolysis under fasting conditions and protects mice against DIO and associated metabolic deficits.

Regulation of lipolysis occurs in response to fluctuating metabolic conditions and nutrient intake and is mediated by hormonal and biochemical signals. Several GPCRs have been identified that play a role in regulating the rate of adipocyte lipolysis (21). Fasting triggers lipolysis and elevated plasma FFA and glycerol levels to provide substrates for oxidative metabolism in other metabolically important tissues. For example, the activation of adipocyte G_s_-coupled β-adrenergic receptors results in cAMP-mediated activation of PKA, resulting in the phosphorylation and activation of HSL and enhanced lipolysis. However, β-adrenergic receptor–stimulated lipolysis is impaired in obesity (5). Stimulation of adipocyte G_i_-coupled receptors has been shown to mediate antilipolytic effects via decrease in cAMP levels (21). In this study, we showed that stimulation of the G_i_-coupled P2Y_14_R decreased adipocyte cAMP levels. Further, P2Y_14_R activation resulted in reduced lipolysis due to decreased phosphorylation of lipolytic enzymes (ATGL and HSL). In vivo lack of P2Y_14_R specifically in adipocytes resulted in increased plasma FFA levels in the fasting state, with no effect on FFA levels observed in freely fed mice. In agreement with this observation, HFD adipo-P2Y_14_^Δ/Δ^ mice showed enhanced phosphorylation of ATGL and HSL only under fasting conditions. Interestingly, fasting induced the downregulation of P2Y_14_R expression levels in eWAT and BAT of HFD mice. These data suggest that the downregulation of P2Y_14_R in WAT during fasting is essential for the activation of lipolytic enzymes, thus stimulating lipolysis.

DIO increased the expression of P2Y_14_R in mouse WAT, indicative of a potential role of this receptor in the development of obesity. Moreover, plasma UDP-glucose levels were increased in obese mice (34), indicating enhanced activity of P2Y_14_R in obese mice. In agreement with this concept, adipocyte specific P2Y_14_R KO mice were protected from DIO, indicating that adipocyte P2Y_14_R deficiency prevented fat accumulation by enhancing lipolysis. Decreased adiposity greatly improved glucose tolerance and insulin sensitivity in HFD adipo-P2Y_14_^Δ/Δ^ mice. Xu et al. reported that whole-body HFD P2Y_14_R null mice showed improved insulin sensitivity in muscle, liver, and adipose tissue, but they did not observe any changes in overall fat mass (34). The authors further reported reduced infiltration of immune cells in liver of P2Y_14_R null mice (34). In this study, we observed marked reduced inflammation in eWAT of adipo-P2Y_14_^Δ/Δ^ mice. Interestingly, HFD adipo-P2Y_14_^Δ/Δ^ mice displayed a modest increase in oxygen consumption and total energy expenditure. This observation agrees with recent findings that elevated plasma FFA levels can promote FA oxidation, thus increasing energy expenditure and providing protection against obesity (31–33). Our data are consistent with a study showing that mice lacking G_i_-coupled CB_1_ receptor showed reduced adiposity, improved insulin sensitivity, and enhanced energy expenditure (12). Surprisingly, enhanced lipolysis due to the lack of adipocyte P2Y_14_R did not result in ectopic lipid deposition in the liver. In contrast, liver weight and triglyceride content were reduced in adipo-P2Y_14_^Δ/Δ^ mice, leading to improved hepatic insulin sensitivity. Taken together, these data demonstrate that the lack of adipocyte P2Y_14_R increased lipolysis in the fasting state and protected against DIO, insulin resistance, adipose inflammation, and liver steatosis.

G_i_-coupled receptors such as GPR84 and CB_1_ have been shown to stimulate the secretion of adipokines such as adiponectin (12, 35, 36). In our study, we observed a marked increase in mRNA expression and circulating levels of adiponectin in HFD adipo-P2Y_14_^Δ/Δ^ mice. Further, treatment of HFD WT mice with P2Y_14_R agonist reduced plasma adiponectin levels. Adiponectin is an antidiabetic adipokine, which has an insulin sensitizing effect on different metabolic tissues (37) and can prevent the development of hepatic steatosis (38). Together, these data suggest that adipocyte P2Y_14_R deficiency enhances circulating levels of adiponectin that contribute to improved systemic insulin sensitivity. In addition, enhanced adiponectin levels may contribute to the observed protection against hepatic lipid accumulation displayed by HFD adipo-P2Y_14_^Δ/Δ^ mice, thereby improving metabolism in HFD adipo-P2Y_14_^Δ/Δ^ mice.

**Figure 7:**
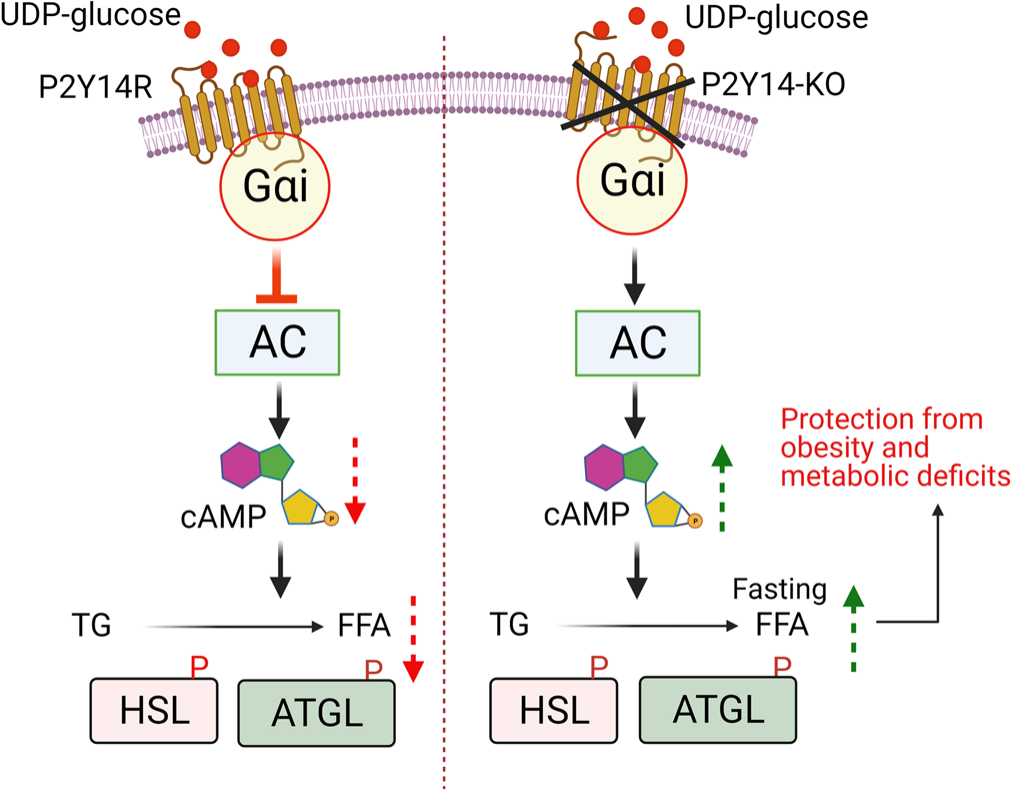
Molecular mechanism for the P2Y_14_R function in white adipocytes. Activation of Gi-coupled P2Y_14_R in adipocytes inhibits lipolysis via decreasing cAMP levels resulting in decreased phosphorylation and activity of lipolytic enzymes. Adipocyte-specific KO of P2Y_14_R causes increase in cAMP levels, increasing phosphorylation and activity of lipolytic enzymes, enhancing lipolysis in fasting conditions. Mice lacking P2Y_14_R in adipocytes were protected from obesity and associated metabolic deficits on high fat diet. P2Y, purinergic; AC, adenylate cyclase; cAMP, cyclic adenosine monophosphate; HSL, hormone-sensitive lipase; ATGL, adipose triglyceride lipase; TG, triglycerides; FFA, free fatty acid; P, phosphorylation.

A recent study reported that the lack of G_i_ signaling in adipocytes leads to a chronic increase (fed and fasted) in lipolysis, resulting in the development of insulin resistance and impaired glucose tolerance on an obesogenic diet (39). In this study, we identified a pathway where the lack of the G_i_-coupled P2Y_14_R specifically in adipocytes led to an increase in lipolysis only in the fasting state, thus protecting mice against DIO and improving glucose tolerance and insulin sensitivity. HFD adipo-P2Y_14_^Δ/Δ^ mice further displayed reduced inflammation, hepatic steatosis, increased adiponectin levels, and enhanced energy expenditure (Figure 7).

P2Y_14_R is predominantly activated by UDP-glucose and other nucleotide sugars (17, 40). Recently, a study revealed that P2Y_14_R can also be potently activated by UDP (41), a nucleotide diphosphate that is a native agonist for Gq-coupled P2Y_6_R (42). Hence, because both receptors share a common agonist, it would be interesting to understand their role in regulating adipocyte function and whole-body glucose and lipid homeostasis. A recent study from our lab demonstrated that mouse adipocytes express functional P2Y_6_R (43). KO of P2Y_6_R specifically in adipocytes protected mice from DIO and decreased systemic inflammation, improving whole-body glucose metabolism. Mechanistically, the lack of P2Y_6_R in adipocytes decreased JNK activation and increased expression and activity of PPARα (43). Interestingly, the lack of P2Y_14_R specifically in adipocytes also protected mice from DIO, improving whole-body metabolism. However, P2Y_14_R is a Gi-coupled receptor and the lack of the receptor enhanced adipocyte lipolysis (fasting) and adiponectin levels in vivo. KO of P2Y_6_R or P2Y_14_R in adipocytes protected mice from the development of liver steatosis associated with obesity. Taken together, these studies suggest that the development of dual antagonist(s) for these receptors or combination therapy with antagonists of both receptors may prove useful in treatment of obesity and T2D.

P2Y_14_R antagonists have been under development for pulmonary diseases and acute kidney injury (44), as well as — potentially — pain and other inflammatory conditions (45). Known antagonists have demonstrated favorable preclinical safety. In this study, acute treatment of mice with P2Y_14_R antagonist prodrug (MRS4741) showed a trend in the decrease in plasma FFA levels caused by treatment of mice with P2Y_14_R agonist (MRS2905). These data show that P2Y_14_R antagonist mimicked the effect of genetic loss of P2Y_14_R in adipocytes and may have been used to enhance receptor-mediated lipolysis, thus providing protection against obesity. Treatment of metabolic conditions with a P2Y_14_R antagonist would have the added benefit of improving β cell function including promoting the release of insulin (46). These findings provide a rational basis for the development of P2Y_14_R antagonists for the treatment of obesity and T2D.

## Methods

### Mouse models.

To generate mice lacking P2Y_14_R selectively in adipocytes, we crossed P2Y_14_R-floxed mice (NIEHS, NIH, Research Triangle Park, North Carolina) with *adipoq-Cre* mice expressing recombinase under the control of adiponectin promoter. *Adipoq-Cre* mice were purchased from the Jackson Laboratories (stock no. 010803; genetic background: C57BL/6J). Mice used for experiments were *P2Y_14_^fl/fl^* (control) and *adipoq-Cre^+/–^P2Y_14_^fl/fl^* (adipo-P2Y_14_^Δ/Δ^) mice. Male mice were used for all experiments. Mice were euthanized using a CO_2_ chamber as required.

### Mouse maintenance and DIO.

Adult mice were used for all experiments reported in this study. Mice were kept on a 12-hour light and 12-hour dark cycle. Animals were maintained at room temperature (23°C) on standard chow (7022 NIH-07 diet, 15% kcal fat, energy density 3.1 kcal/g; Envigo). Mice had free access to food and water. To induce obesity, groups of mice were switched to an HFD (F3282, 60% kcal fat, energy density 5.5 kcal/g; Bio-Serv) at 8 weeks of age. Mice consumed the HFD for at least 8 weeks, unless stated otherwise.

### Human subcutaneous adipose tissue.

The human adipose samples used in this study were described in a previous study by Pydi et al. (47). Briefly, subcutaneous adipose tissues were obtained from the abdominal area of human subjects under local anesthesia using an aspiration needle. RNA was isolated from fat tissues and gene expression analysis was performed as described above.

### In vivo metabolic phenotyping.

Glucose tolerance tests (GTTs) were carried out on mice after a 12-hour overnight fast. After checking fasted blood glucose levels, animals were injected i.p. with glucose (1 or 2 g/kg as indicated), and blood was collected from the tail vein at 15, 30, 60, and 120 minutes after injection. Blood glucose concentrations were determined using a Contour portable glucometer (Bayer). Insulin tolerance tests (ITTs) were also carried out on mice fasted overnight for 12 hours. Fasted blood glucose levels were determined, and then mice were injected i.p. with human insulin (0.75 or 1 U/kg; Humulin, Eli Lilly) as indicated. Blood glucose levels were determined as described above for GTT. All metabolic tests were performed with adult mice that were at least 8 weeks old.

### Body composition analysis.

Mouse body mass composition (lean vs. fat mass) was measured using a 3-in-1 Echo MRI Analyzer (Echo Medical System).

### RNA extraction and gene expression analysis.

Dissected tissues were frozen immediately on dry ice. Total RNA from tissues was extracted using the RNeasy Mini Kit (QIAGEN). Total RNA (500 ng of RNA) was converted into cDNA using SuperScript III First-Strand Synthesis SuperMix (Invitrogen, Thermo Fisher Scientific). Quantitative PCR was performed using the SYBR green method (Applied Biosystems, Thermo Fisher Scientific). Gene expression was normalized to the expression of 18s rRNA using the ΔΔCt method. The primer sequences used in this study are provided in Supplemental Table 1.

### Plasma metabolic profiling.

Blood was collected from the tail vein of mice in chilled K_2_-EDTA–containing tubes (RAM Scientific). Blood was collected from a group of mice that had free access to food (fed state) or from overnight fasted mice. Blood was centrifuged at 4°C for 10 minutes at 10,000*g* to obtain plasma. ELISA kit (Crystal Chem) was used to measure plasma insulin levels, following the manufacturer’s instructions. Leptin and adiponectin levels were measured using ELISA kits from R&D Systems, Bio-Techne. Plasma FFA levels were determined using a commercially available kit (MilliporeSigma).

### Isolation, culture, and differentiation of white adipocytes.

White preadipocytes were isolated, cultured, and differentiated as described previously (43). Excised fat depot was minced into small pieces and digested at 37°C for 45 minutes in Krebs-Ringer-Hepes-bicarbonate buffer (KRH buffer: 120 mM NaCl, 3.6 mM KCl, 0.5 mM NaH_2_PO_4_, 0.2 mM MgSO_4_, 1.5 mM CaCl_2_, 10 mM Hepes [pH 7.4], 2 mM NaHCO_3_) containing 3.3 mg/mL collagenase I (MilliporeSigma). Other chemicals were obtained as reagent grade (MilliporeSigma), unless noted. After digestion, 10 mL of KRH buffer was added, and the cell suspension was filtered through a 70 μm cell strainer, followed by centrifugation at 700*g* for 5 minutes at room temperature. The supernatant was discarded, and the cell pellet was resuspended in KRH buffer and recentrifuged to obtain a cell pellet of mesenchymal stem cells. The pellet was resuspended in DMEM containing 10% FBS and 1% penicillin-streptomycin (pen-strep). Approximately 80,000 cells were seeded per well of collagen I–coated 12-well plates (Corning) and cultured at 37°C, 10% CO_2_. Cells were allowed to reach 100% confluence by replenishing media every second day. Two days after reaching confluence, cells were induced for differentiation using DMEM supplemented with 10% FBS, 1% pen-strep, 0.5 μM insulin, 250 μM 3-isobutyl-1-methylxanthine (IBMX), 2 μM troglitazone, 0.5 μM dexamethasone, and 60 μM indomethacin. After 72 hours’ exposure to the induction media, cells were incubated in differentiation DMEM media supplemented with 10% FBS, 1% pen-strep, and 0.5 μM insulin for the next 72 hours. Mature differentiated white adipocytes were used for further studies.

### Isolation of mature adipocytes.

The isolation of mature mouse adipocytes was performed as described previously (47). In brief, mouse fat pads were collected and digested with KRH buffer containing collagenase 1 (3 mg/mL). Digested tissues were filtered through a 250 μm cell strainer. After a 5-minute centrifugation step at 60*g*, the top layer containing mature adipocytes was collected and used for further experiments.

### cAMP assay.

Differentiated primary white adipocytes were washed twice with DPBS and serum starved in incomplete DMEM for 2 hours. Cells were incubated with agonist MRS2905 (2 nM, Tocris, Bio-Techne) for 30 minutes in DMEM+IBMX (100 μM) as indicated at 37°C. Subsequently, cells were treated with CL-316,243 (10 nM) for 30 minutes as indicated. Cells were lysed using 0.05N HCl, and intracellular cAMP levels were quantified using a detection kit from Cisbio Bioassays.

### Lipolysis assay.

Differentiated primary white adipocytes were washed twice with DPBS, followed by serum starvation in DMEM for 2 hours at 37°C. Cells were stimulated with P2Y_14_R agonist (MRS2905, 2 nM) for 30 minutes, followed by treatment with CL-316,243 (10 nM) for another 30 minutes, as indicated. In another set of experiments, cells were treated with vehicle or PPTN (200 nM; Tocris, Bio-Techne) for 30 minutes, followed by insulin (10 nM) for 15 minutes as indicated. Cells were then stimulated with CL-316,243 (10 nM) for another 30 minutes, as indicated. To terminate the reaction, the cell culture plates were incubated on ice, and cell media were collected for the measurement of glycerol levels using a glycerol assay kit (MilliporeSigma). Cells were lysed in RIPA buffer, and protein concentrations were determined for each well. Glycerol levels were normalized to protein levels.

### In vivo insulin signaling.

HFD adipo-P2Y_14_^Δ/Δ^ and control mice were fasted for 4 hours. Mice were then anesthetized using isoflurane, and the abdominal cavity exposed to access the inferior vena cava. Subsequently, 5 U of human insulin (Humulin, Eli Lilly) dissolved in 100 μL of 0.9% saline was injected into the vena cava (48). Tissues including liver, adipose, and skeletal muscle were harvested 5–10 minutes after injection and snap-frozen in liquid nitrogen for Western blot studies.

### Treatment of mice with MRS2905 and/or MRS4741.

Adipo-P2Y_14_^Δ/Δ^ and control mice were fasted for 12–14 hours. Mice were injected with MRS2905 (10 mg/kg, i.p.) or saline, and blood was collected from the tail vein at 0, 45, and 180 minutes after injection. In another set of experiments, mice were injected with P2Y_14_R antagonist (MRS4741 [2-(dimethylamino)-2-oxoethyl 4-(4-[1-acetylpiperidin-4-yl]phenyl)-7-(4-[trifluoromethyl]phenyl)-2-naphthoate]); 20 mg/kg, i.p., synthesized as reported in ref. 24; vehicle was DMSO/Kolliphor EL/PBS, 15:15:70 by volume) in the evening before food removal. In the morning, mice were injected with a second dose of MRS4741 (20 mg/kg, i.p.). After 2 hours, mice were injected with P2Y_14_R agonist, MRS2905 (10 mg/kg, i.p.). Blood was collected from the tail vein immediately before and 45 minutes after agonist injection. Plasma was obtained by centrifuging blood samples at 10,000*g* for 10 minutes at 4°C. Plasma FFA levels were determined using the Fujifilm NEFA kit. Adiponectin levels were measured using a kit from R&D Systems, Bio-Techne.

### Liver triglyceride content.

Liver triglyceride levels were measured by homogenizing 20 mg of hepatic tissue in PBS. A chloroform/methanol (2:1) mixture was then added to the liver homogenate. The homogenate was centrifuged, and the organic phase was transferred to a new tube and dried overnight. Each sample was dissolved in ethanol containing 1% Triton X-100, and triglyceride levels were measured using a triglyceride reagent (MilliporeSigma). Triglyceride levels were normalized to protein levels in liver homogenates.

### Western blot studies.

Western blot studies were carried out as described previously (43). Briefly, adipocytes or adipose tissues were homogenized in adipocyte lysis buffer (50 mM Tris pH 7.4, 500 mM NaCl, 1% NP-40, 20% glycerol, 5 mM EDTA, and 1 mM phenylmethylsulfonyl fluoride) supplemented with EDTA-free protease inhibitor cocktail and phosphatase inhibitors cocktail (Roche). Protein concentrations in the lysates were determined using a BCA Protein Assay Kit (Pierce, Thermo Fisher Scientific). Protein was denatured in NuPAGE LDS sample buffer (Thermo Fisher Scientific) and β-mercaptoethanol at 90°C for 5 minutes. Protein lysates were separated using 4%–12% SDS-PAGE (Invitrogen, Thermo Fisher Scientific) and transferred to nitrocellulose membranes (Bio-Rad). Membranes were incubated with primary antibody overnight at 4°C in 5% w/v BSA prepared in 1x TBS with 0.1% Tween 20. On the next day, the membranes were washed and incubated with HRP-conjugated anti-rabbit/mouse secondary antibody. SuperSignal West Pico Chemiluminescent Substrate (Pierce, Thermo Fisher Scientific) was used to visualize immunoreactive bands using an Azure Imager C600 (Azure Biosystems). Images were analyzed using NIH ImageJ software. A list of primary and secondary antibodies used are provided in Supplemental Table 2.

### Histology of fat tissues and liver.

Mouse liver and fat depots were isolated and frozen in liquid nitrogen or immersed in 4% paraformaldehyde. Tissue samples were sectioned and stained using standard techniques. Fixed adipose tissues were stained for F4/80 using standard IHC methods. Liver sections were subjected to oil red O or H&E staining. The stained images were visualized and captured using a BZ-9000 Microscope (Keyence).

### Indirect calorimetry.

Energy expenditure (O_2_ consumption/CO_2_ production) and food intake were measured in mice housed at 22°C using an Oxymax-CLAMS monitoring system (Columbus Instruments). Before subjecting mice to HFD feeding, mice were maintained on RC for 3 days. On day 4, mice started to consume the HFD. Data were collected every 4 minutes. Mice were maintained on HFD for 4 additional days before terminating the experiment. Water and food were provided ad libitum. Data were analyzed using ANCOVA (27).

### Data and materials availability.

All data needed to evaluate the conclusions in the paper are present in the paper and/or Supplementary Figures 1–7. Additional data related to this paper may be requested from the authors.

### Statistics.

Data are expressed as mean ± SEM for the number of observations indicated. Data were tested by 2-way ANOVA, followed by post hoc tests, or by 2-tailed unpaired Student’s *t* test, as appropriate. A *P* value of less than 0.05 was considered statistically significant.

### Study approval.

All animal studies were carried out according to the *Guide for the Care and Use of Laboratory Animals* (National Academies Press, 2011) and were approved by the NIDDK Institutional Animal Care and Use Committee, protocol K083-LBC-20.

## Author contributions

SJ and KAJ conceived the experimental design. SJ, SPP, ELK, and OG performed the experiments. YHJ, MS, TPK, DNC, and JW contributed the research materials. SJ, SPP, OG, JW, and KAJ analyzed the data and wrote the manuscript. SJ wrote the first draft.

## Supplementary Material

Supplemental data

## Version 1 05/24/2021

Electronic publication
