# Supplementary material for: Adipocyte P2Y14 receptors play a key role in regulating whole-body glucose and lipid homeostasis
Source: JCI Insight. 2021 May 24;6(10):e146577. doi: 10.1172/jci.insight.146577 (PMC8262345; doi:10.1172/jci.insight.146577)
Supplement: Supplemental data [file jciinsight-6-146577-s207.pdf]

Supplemental Information for

Adipocyte P2Y$_{14}$ receptors play a key role in regulating whole-body glucose and lipid homeostasis

Shanu Jain, Sai P. Pydi, Young-Hwan Jung, Mirko Scortichini, Efrat L. Kesner, Tadeusz P. Karcz, Donald N. Cook, Oksana Gavrilova, Jürgen Wess, Kenneth A. Jacobson

corresponding authors: Shanu Jain, Kenneth A. Jacobson

Email: shanu.jain@nih.gov, kennethj@niddk.nih.gov

This PDF file includes:

Figures S1 to S7

Tables S1 to S2

Supplementary Figures

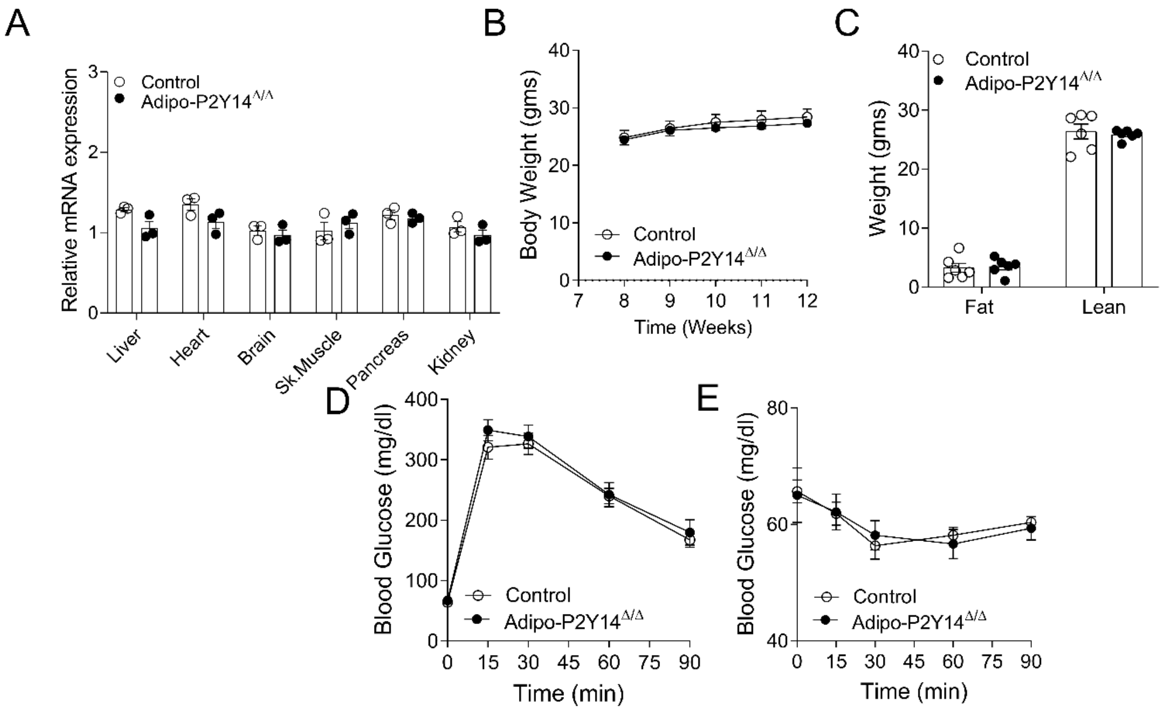

Figure S1: Metabolic analysis of adipo-P2Y14$^{\Delta/\Delta}$ and control mice maintained on chow diet (RC).

(A) mRNA levels of $P2Y_{14}R$ in liver, heart, brain, skeletal muscle, pancreas, and kidney of adipo-P2Y14$^{\Delta/\Delta}$ and control mice on RC (n=3/group).
(B) Body weight measurements on RC (n=6-8/group).
(C) Body composition of mice maintained on RC (n=6/group).
(D) Glucose tolerance test (IGTT, 2 g/kg glucose i.p.) (n=12-14/group).
(E) Insulin tolerance test (ITT, 0.75 U/kg insulin i.p.) (n=6/group).

All data are expressed as means ± SEM. (A, C: two-tailed Student's t-test; B, D-E: two-way ANOVA followed by Bonferroni's post hoc test).

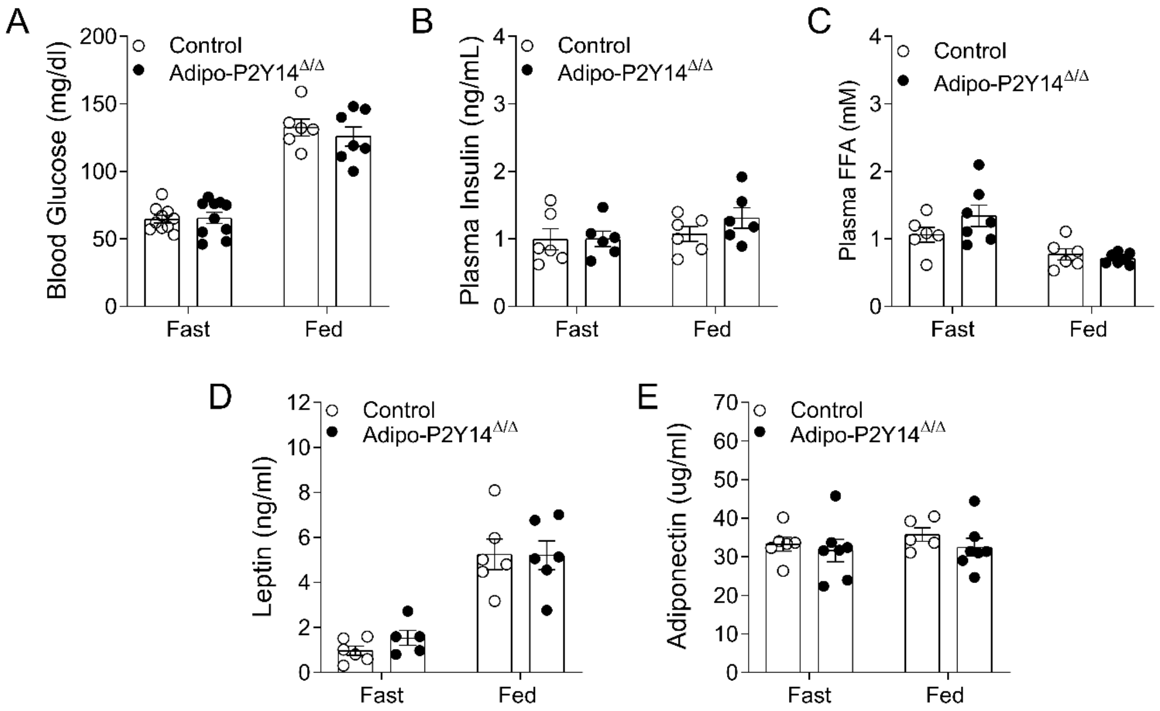

Figure S2: Plasma profiling of adipo-P2Y14$^{\Delta/\Delta}$ and control mice maintained on chow diet (RC).

(A) Fasting and fed blood glucose levels (n=6-10/group).
(B) Fasting and fed plasma insulin levels (n=6/group).
(C) Fasting and fed plasma free fatty acid (FFA) levels (n=6 or 7/group).
(D) Fasting and fed plasma leptin levels (n=5 or 6/group).
(E) Fasting and fed plasma adiponectin levels (n=5-7/group).

All data are expressed as means ± SEM. (two-tailed Student's t-test).

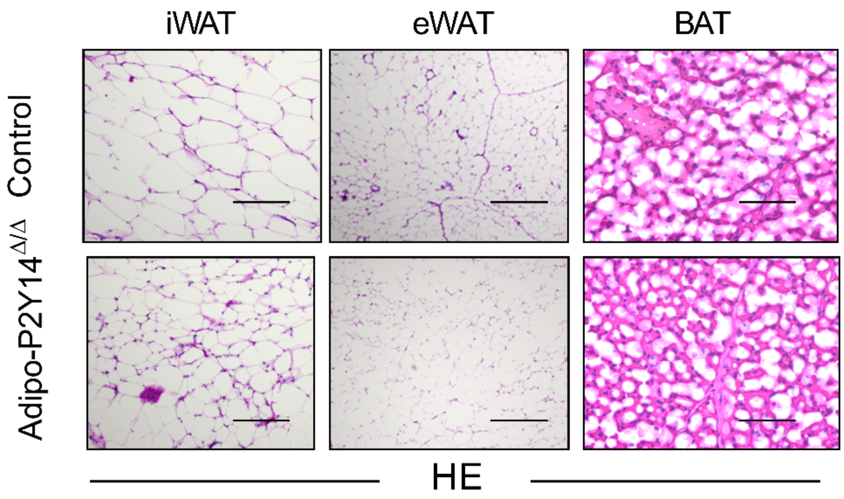

Figure S3: Reduced adipocyte size in WAT of adipo-P2Y14$^{\Delta/\Delta}$ mice on HFD.

Representative H&E stained sections of iWAT, eWAT and BAT from HFD adipo-P2Y14$^{\Delta/\Delta}$ and control mice. All experiments were conducted on mice fed on HFD for at least 8 weeks.

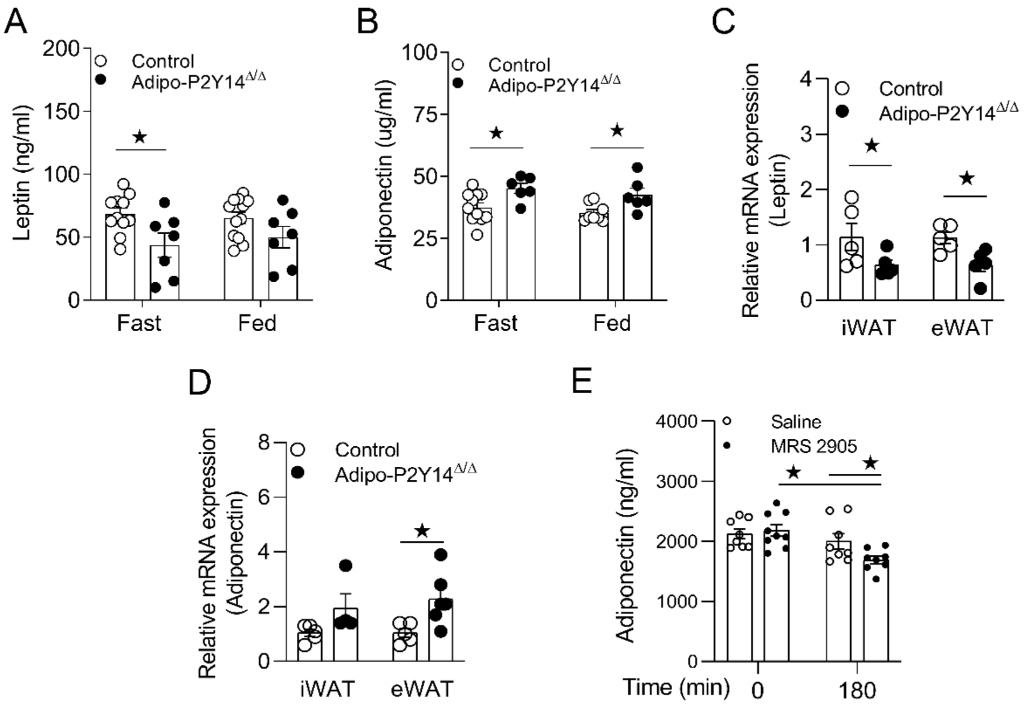

Figure S4: Leptin and adiponectin levels adipo-P2Y14$^{\Delta/\Delta}$ mice on HFD.

(A) Fasting and fed plasma leptin levels (n=7-11/group).

(B) Fasting and fed plasma adiponectin levels (n=6-12/group).

(C) mRNA expression levels of leptin in iWAT and eWAT of HFD adipo-P2Y14$^{\Delta/\Delta}$ and control mice (n=4-6/group).

(D) mRNA expression levels of adiponectin in iWAT and eWAT of HFD adipo-P2Y14$^{\Delta/\Delta}$ and control mice (n=4-6/group).

(E) Plasma adiponectin levels in HFD WT mice i.p. injected with saline or MRS2905 (10 mg/kg, i.p.). Blood was collected immediately before and 180 min after injections (n=8 or 9/group).

All data are expressed as means ± SEM. *p<0.05 (A-D: two-tailed Student's t-test; E: One-way ANOVA followed by Bonferroni's post hoc test). All experiments were conducted on mice fed on HFD for at least 8 weeks.

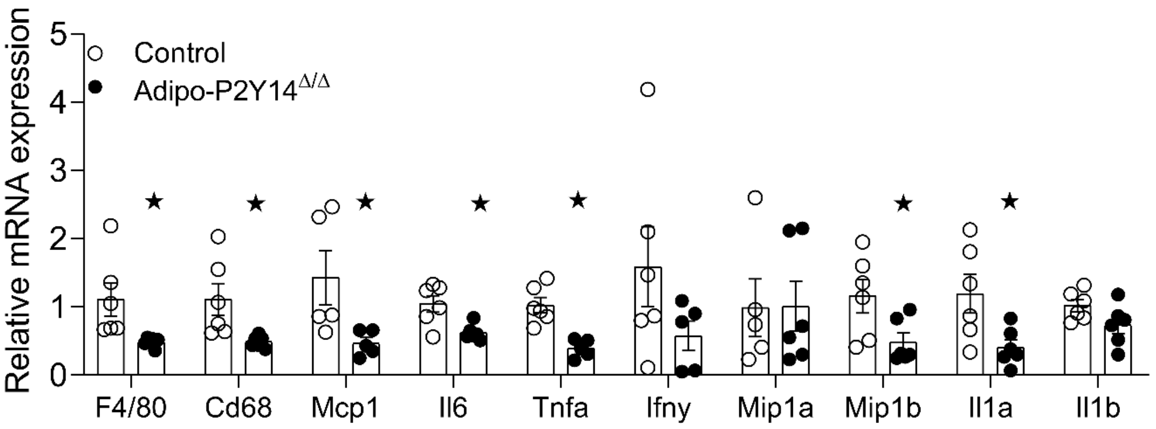

Figure S5: Reduced inflammation in BAT of adipo-P2Y14$^{\Delta/\Delta}$ mice on HFD.

Relative mRNA expression levels of inflammatory genes in BAT from HFD adipo-P2Y14$^{\Delta/\Delta}$ and control mice (n=5-6/group).

The expression of 18sRNA was used to normalize qRT-PCR data. All data are expressed as means ± SEM. *$p < 0.05$ (two-tailed Student's t-test). All experiments were conducted on mice fed on HFD for at least 8 weeks.

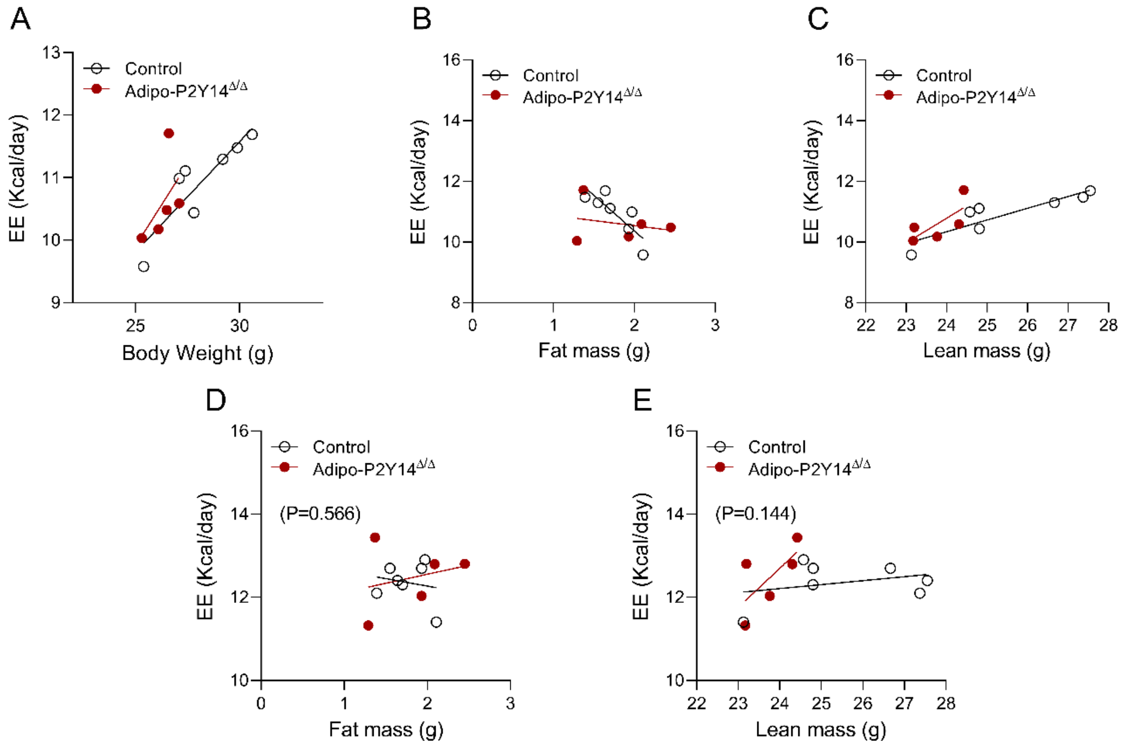

Figure S6: Energy expenditure in RC and HFD adipo-P2Y14$^{\Delta/\Delta}$ mice.

(A) Energy expenditure normalized to body weight on RC.
(B) Energy expenditure normalized to fat mass on RC.
(C) Energy expenditure normalized to lean mass on RC.
(D) Energy expenditure normalized to fat mass on HFD.
(E) Energy expenditure normalized to lean mass on HFD.

For all panels, n=5-7/group. Data were analyzed using ANCOVA.

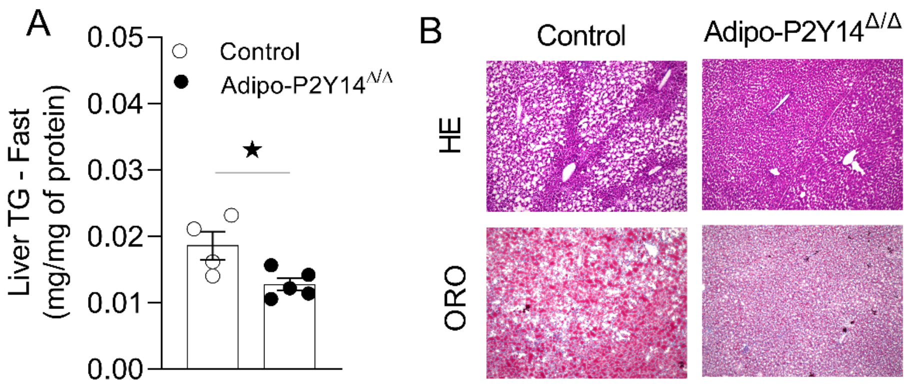

Figure S7: Reduced liver steatosis in fasted HFD adipo-P2Y14$^{\Delta/\Delta}$ mice.

(A) Liver triglyceride levels in fasted control and adipo-P2Y14$^{\Delta/\Delta}$ mice. (n=4-5/group).
(B) ORO and HE stain in liver sections from fasted control and adipo-P2Y14$^{\Delta/\Delta}$ mice.

All data are expressed as means ± SEM. *p< 0.05 (two-tailed Student's t-test). All experiments were conducted on mice fed on HFD for at least 8 weeks.

Supplementary Tables

Table S1: List of primers used for RT-PCR studies.

Gene name	Forward (5'-3')	Reverse (5'-3')
P2ry14	AGCAGATCATTCCCGTGTTGT	AGCCACCACTATGTTCTTGAGA
18S rRNA	CGGCTACCACATCCAAGGAA	GCTGGAATTACCGCGGCT
Leptin	CAAGCAGTGCCTATCCAGA	AAGCCCAGGAATGAAGTCCA
Adiponectin	GCACTGGCAAGTTCTACTGCAA	GTAGGTGAAGAGAACGGCCTTGT
F4/80	TCCTGCTGTGTCGTGCTGTTC	GCCGTCTGGTTGTCAGTCTTGTC
Cd68	TGTCTGATCTTGCTAGGACCG	GAGAGTAACGGCCTTTTTGTGA
Mcp1	GCTCAGCCAGATGCAGTTAA	TCTTGAGCTTGGTGACAAAAACT
Il6	TAGTCCTTCCTACCCCAATTTCC	TTGGTCCTTAGCCACTCCTTC
Tnfa	CCCTCACACTCAGATCATCTTCT	GCTACGACGTGGGCTACAG
Ifng	CGGCACAGTCATTGAAAGCCTA	GTTGCTGATGGCCTGATTGTC
Mip1a	TGAGAGTCTTGGAGGCAGCGA	TGTGGGTACTTGGCAGCAAACA
Mip1b	AACAACATGAAGCTCTGCGT	AGAAACAGCAGGAAGTGGGA
IL1a	ACGTCAAGCAACGGGAAGAT	AAGGTGCTGATCTGGGTTGG
IL1b	CTCCACCTCAATGGACAGAA	GCCGTCTTTCATTACACAGG
Srebp1	AGTGGCAAAGGAGGCACTAC	CACCCTCTGGAAGACCACA
Fas	GGAGGTGGTGATAGCCGGTAT	TGGGTAATCCATAGAGCCCAG
Fgf21	CTGCTGGGGGTCTACCAAG	CTGCGCCTACCACTGTTCC
G6pc	CGACTCGCTATCTCCAAGTGA	GTTGAACCAGTCTCCGACCA
Pdk4	CCGCTTAGTGAACACTCCTTC	TGACCAGCGTGTCTACAAACT
Pck1	CTGCATAACGGTCTGGACTTC	CAGCAACTGCCCGTACTCC
Serca1	TGTTTGTCCTATTTCGGGGTG	AATCCGCACAAGCAGGTCTTC
Serca2	GAGAACGCTCACACAAAGACC	CAATTCGTTGGAGCCCCAT
Universal-Cre	ACCTGAAGATGTTCGCGATTATCT	ACCGTCAGTACGTGAGATATCTT

Table S2: List of antibodies used for Western blot studies.

Antibodies	Source[a]	Catalog #
T-HSL	Cell Signaling	4107
p-HSL (Ser563)	Cell Signaling	4139
T-ATGL	Abcam	2138
p-ATGL (Ser406)	Abcam	ab135093
T-AKT	Cell Signaling	9272
p-AKT (Thr308)	Cell Signaling	2965
p-AKT (Ser473)	Cell Signaling	4060
T-GSK-3β	Cell Signaling	5676
p-GSK-3β (Ser9)	Cell Signaling	9336
β-Actin	Cell Signaling	4970

[a] Cell Signaling Technology, Danvers, MA, USA, https://www.cellsignal.com/;
Abcam, Cambridge, MA, USA, https://www.abcam.com/.
